# Manganese-Enhanced MRI Reflects Both Activity-Independent and Activity-Dependent Uptake within the Rat Habenulomesencephalic Pathway

**DOI:** 10.1371/journal.pone.0127773

**Published:** 2015-05-26

**Authors:** Leiming Wang, Hanbing Lu, P. Leon Brown, William Rea, Bruce Vaupel, Yihong Yang, Elliot Stein, Paul D. Shepard

**Affiliations:** 1 Neuroimaging Research Branch, National Institute on Drug Abuse, IRP, Baltimore, Maryland, 21224, United States of America; 2 Department of Psychiatry and the Maryland Psychiatric Research Center, University of Maryland School of Medicine, Baltimore, Maryland, 21228, United States of America; Dalhousie University, CANADA

## Abstract

Manganese-enhanced magnetic resonance imaging (MEMRI) is a powerful technique for assessing the functional connectivity of neurons within the central nervous system. Despite the widely held proposition that MEMRI signal is dependent on neuronal activity, few studies have directly tested this implicit hypothesis. In the present series of experiments, MnCl_2_ was injected into the habenula of urethane-anesthetized rats alone or in combination with drugs known to alter neuronal activity by modulating specific voltage- and/or ligand-gated ion channels. Continuous quantitative T1 mapping was used to measure Mn^2+^ accumulation in the interpeduncular nucleus, a midline structure in which efferents from the medial habenula terminate. Microinjection of MnCl_2_ into the habenular complex using a protocol that maintained spontaneous neuronal activity resulted in a time-dependent increase in MEMRI signal intensity in the interpeduncular nucleus consistent with fast axonal transport of Mn^2+^ between these structures. Co-injection of the excitatory amino-acid agonist AMPA, increased the Mn^2+^-enhanced signal intensity within the interpeduncular nucleus. AMPA-induced increases in MEMRI signal were attenuated by co-injection of either the sodium channel blocker, TTX, or broad-spectrum Ca^2+^ channel blocker, Ni^2+^, and were occluded in the presence of both channel blockers. However, neither Ni^2+^ nor TTX, alone or in combination, attenuated the increase in signal intensity following injection of Mn^2+^ into the habenula. These results support the premise that changes in neuronal excitability are reflected by corresponding changes in MEMRI signal intensity. However, they also suggest that basal rates of Mn^2+^ uptake by neurons in the medial habenula may also occur via activity-independent mechanisms.

## Introduction

Manganese (Mn^2+^) is an essential trace element that serves as an electron donor in a variety of enzymatic reactions [1, 2]. Its entry into excitable cells occurs through uptake by heavy metal transporters [2, 3] and limited passage through voltage- and ligand-gated ion channels [4, 5]. In CNS neurons, Mn^2+^ is loaded into vesicles and transported along the axon by fast anterograde transport [6, 7], where it is released at the axon terminal. Mn^2+^ exhibits strong magnetic permeability in the presence of an externally applied magnetic field, slowing the relaxation time constants of tissue water [8, 9], resulting in a significant enhancement in MRI contrast. The ability of Mn^2+^ to trace the flow of information within a neuronal circuit has made manganese-enhanced magnetic resonance imaging (MEMRI) a powerful technique for assessing the functional connectivity of CNS neurons [10–13].

Divalent Mn^2+^ shares several physiochemical properties with Ca^2+^ including a comparable ionic radius and ability to permeate voltage- and ligand-gated Ca^2+^ channels [4, 5, 14]. The established role of Ca^2+^ conductances as mediators of neuronal excitability led to the assertion that Mn^2+^ entry into neurons is activity dependent. In an early and influential study, Lin and Koretsky [15] showed that glutamate enhances MEMRI signal intensity in the cortex after systemic injection of MnCl_2_ and disruption of the blood-brain barrier. Subsequently, regionally-specific enhancement of T1-weighted images following systemic MnCl_2_ were observed in barrel cortex following whisker stimulation [16], in somatosensory cortex following cutaneous stimulation [15, 17, 18], in the mesocorticolimbic system after acute cocaine administration [19], during tonotopic activation of the inferior colliculus [20], and kainic acid-induced activation of rat hippocampus [21]. Collectively, these data are consistent with the notion that MEMRI is driven by an increase in neuronal activity.

Despite the widely held proposition that Mn^2+^ entry into excitable cells is largely or even exclusively dependent on neuronal activity, relatively few studies have systematically examined this implicit hypothesis in CNS neurons [19, 22]. In the present series of experiments, we microinjected MnCl_2_ into the habenula of urethane-anesthetized rats alone and/or in combination with compounds known to modulate specific voltage- and ligand-gated ion channels. Continuous quantitative T1 mapping was used to measure Mn^2+^ accumulation in the interpeduncular nucleus (IPN), a midline structure in which many habenular efferents pass or terminate via the fasciculus retroflexus [23]. To anchor our MRI observations, in a parallel experiment, single unit recording of habenular neurons was used to track firing activity under these same conditions. Taken together, our results indicate that Mn^2+^ enters habenular projection neurons through impulse-dependent and impulse-independent mechanisms and that pharmacologically-induced increases in neuronal activity are associated with increased Mn^2+^ uptake that is both Ca^2+^ and Na^+^-dependent.

## Materials and Methods

### Animals

A total of 71 male Sprague–Dawley rats (250–350 g, Charles River Laboratories, VA) were used in this study. Animals were housed in a temperature controlled vivarium under a 12:12hr light:dark cycle and provided free access to food and water.

### Ethics Statement

The experiments described in this study were carried out in strict accordance with the recommendations in the Guide for the Care and Use of Laboratory Animals of the National Institutes of Health. The protocol was approved by the Animal Care and Use Committee of the National Institute on Drug Abuse-IRP (Animal Study Protocol—08-NRB-22) and the University of Maryland School of Medicine (IACUC Protocol 0914014). All surgery was performed under urethane anesthesia and every effort was made to minimize suffering.

### Intracerebral MnCl_2_ Injection

Rats were anesthetized with urethane (1.3 g/kg, i.p. Sigma-Aldrich Co. USA) and mounted in a stereotaxic apparatus equipped with a feedback controlled heating pad that was used to maintain body temperature at 37°C. The scalp was incised along the midline and a burr hole drilled in the skull overlying the right habenula and the dura carefully incised. Injection cannula were fashioned from a 4 cm length of stainless steel tubing (30-gauge) that was beveled to a 45° angle on one end. The opposite end was inserted into a length of polyethylene tubing (PE-20). The cannula was firmly attached to a piezoelectric microdrive (Burleigh Inchworm, Burlingame, NY) that was used to position it within the lateral habenula (3.5 mm posterior to Bregma, 0.9 mm lateral and 5.3 mm below cortical surface). Prior to positioning, the cannula was backfilled with one or more of the following solutions: MnCl_2_, 105 mM; AMPA, 100 μM; tetrodotoxin (TTX, 2 μM); NiCl_2_, 0.5 mM. Manganese was obtained in solution as a concentrated stock (1 ± 0.01 M, Sigma-Aldrich Co, St. Louis, MO.) and diluted in Tris-HCl to a final pH of 7.35–7.45. The osmolality of the solution was adjusted to 290–300 mOsm using a vapor pressure osmometer (Wescor, Logan, Utah). Solutions were filtered, sterilized, loaded into the injection cannula and attached to a 0.5 μl Hamilton microsyringe. Solutions containing MnCl_2_ were injected in a volume of 30 nl at a rate of 0.5 nl/minute using a programmable syringe pump. This procedure has been shown to minimize the toxicity associated with intracerebral injections of MnCl_2_ [24]. Injection cannula remained in place for 15 minutes to prevent backflow through the needle track. Following removal of the injection cannula, rats were either prepared for extracellular single unit recording study (n = 10) or removed from the stereotaxic and the scalp closed with cotton suture before transfer to the MRI scanner (n = 61).

### Single Unit Recording Studies

Recording electrodes were prepared from borosilicate glass capillary tubing (1.5 mm OD) using a vertical puller, filled with a solution of 1M NaCl saturated with fast green dye and the tips broken back to a final impedance of 10–14 MΩ, in vitro. Electrodes were attached to a piezoelectric microdrive and positioned over the same burr hole that had been used to insert the injection cannula. The electrode was lowered into the region overlying the habenula (AP: 3.5 mm posterior to Bregma) at a 10° angle in the mediolateral plane and advanced at 1–2 um/sec. A total of three passes or “tracks” were made through the dorsoventral extent of the habenula, beginning at 3.9 mm ventral to cortical surface, at mediolateral coordinates corresponding to the medial Hb (MHb: 0.9 mm from the midline), medial aspect of the lateral habenula (LHb(m):1.2 mm from the midline) and lateral aspect of the lateral habenula (LHb(l): 1.5 mm from the midline). Track sequence (medial to lateral vs. lateral to medial) was randomly assigned to each rat. Electrode potentials were amplified, filtered (0.1–8 kHz bandpass), and monitored in real time using a digital oscilloscope and audiomonitor. Well isolated spikes (signal:noise > 5:1) were digitized at 20 kHz using a 16-bit laboratory interface (Digidata 1321A; Molecular Devices, Union City, CA) and stored on disk for analysis offline with the Spike 2 software package (CED, Manchester, UK). Spontaneous activity was recorded for up to two minutes and the depth of each unit recorded before advancing the electrode in search of another cell. At the end of each track, fast green was iontophoretically ejected from the pipette tip by application of a -25 μA DC current to the recording electrode for 30 minutes.

At the conclusion of the recording study, rats were deeply anesthetized with urethane and perfused transcardially with 60 ml of phosphate buffered saline followed by 60 ml of 10% formalin (pH 7.4, 4°C) and the brain removed and post-fixed overnight. Contiguous 40 μm thick coronal sections though the habenula were prepared using a cryostat and sections containing dye spots were slide mounted, and counterstained with 0.1% neutral red. Low power photomicrographs were obtained using a Zeiss Axioplan microscope equipped with an Olympus DP70 digital camera and overlayed on corresponding sections obtained from the Paxinos and Watson rat brain atlas [25] to reconstruct the trajectory of each track and the position of each recorded cell. Only tracks that passed through the full dorsoventral extent of the habenula were included in the analysis.

### *In Vivo* MRI

MEMRI imaging was performed using a Bruker Biospin 9.4T scanner (Bruker, Karlsruhe, Germany) equipped with an active-shielded gradient coil. The inner diameter of the gradient coil was 0.12 m, and maximum gradient strength was 40 mT/m. A birdcage coil driven in linear mode was used for RF excitation, and a single-turn circular surface coil (2.5 cm in diameter) was used for signal reception. Immediately following closure of the injection site wound margin, rats were intubated and placed in a customized holder that stabilized the head and provided feedback-controlled regulation of body temperature. Respiratory and cardiac rate were continuously monitored Small Animal Instruments, Inc, New York, USA).

The distribution of Mn^2+^-induced signal in the brain was dynamically quantified using a FLASH-based inversion-recovery Look-Locker sequence. This sequence was capable of mapping T1 values with high spatial resolution. Scan parameters were: inversion time (TI) interval = 180.15 ms with a total of 25 inversion times, flip angle = 15°, echo time (TE) = 3.5 ms, number of averages = 2, field of view (FOV) = 28 × 28 mm^2^, matrix size = 96 × 96, 11 slices with a slice thickness of 1 mm. Images were reconstructed to 128 × 128 by zero-padding. Each scan lasted for 15 min. The first scan was performed 135 minutes following the start of the Mn^2+^ injection. T1 data were acquired every 30 minutes for the duration of the experiment (up to 7 hours). Identification of the injection site was performed using the decussation of the anterior commissure as an anatomical landmark (-0.36 mm from bregma), which appears dark in T2-weighted anatomical images and could be readily identified [19]. Similar to the study by Bock et al, [26], in addition to performing T1-mapping, we also acquired T1-weighted images using a traditional spin echo sequence (TR = 450 ms. TE = 8 ms, FOV 28 × 28 mm^2^, matrix size = 96 × 96). The enhancement of MRI signal as a result of Mn^2+^ accumulation can be readily identified in these T1-weighted images.

### Data Processing

T1 values were derived in two steps: first, the inversion-recovery signal was fitted with a three-parameter single-exponential model [27]:

S=|A+B×exp(−TI/T1*)|, (1)

Here, *S* is the signal intensity at a particular inversion time (TI). *A*, *B* and T1* are the 3 parameters to be fitted to the inversion-recovery curve. Here T1* is apparent longitudinal relaxation time, and 1/T* = 1/*T*1- ln(cosθ)/*TR*, where *θ* is the flip angle and *TR* is the repetition time [28], both remained constant across the experiments. A customized program was written within the AFNI software framework [29] to perform the curve fitting on a voxel-wise basis. Previous studies [30, 31] have shown that the relationship between the concentration of Mn^2+^ in brain tissue and corresponding T1 values can be modeled as:

$$1/\text{T}1\left(\left[{\text{Mn}}^{2+}\right]\right)\;=\;1/\text{T}1\left(0\right)\;+\;\beta \times \left[{\text{Mn}}^{2+}\right]$$ (2)

Where [Mn^2+^] indicates the concentration of manganese, 1/T1([Mn^2+^]) indicates the longitudinal relaxation rate at a given concentration [Mn^2+^], and β is the longitudinal relaxivity constant. Since our study aimed to determine the dynamic distribution of Mn^2+^ in brain following intracerebral micro-injection, we report longitudinal relaxation rate (R1 = 1/T1) across time, which is linearly related to [Mn^2+^], as shown in Eq (2).

### Statistical analysis

All numerical data are presented as mean ± standard error (SEM). For MRI data, comparisons between the control and experiment groups were made using two-way repeated measures ANOVA. A one-way ANOVA was used to compare the electrophysiological endpoints between habenular subnuclei. Significance levels were set at p < 0. 05.

## Results

**Fig 1:**
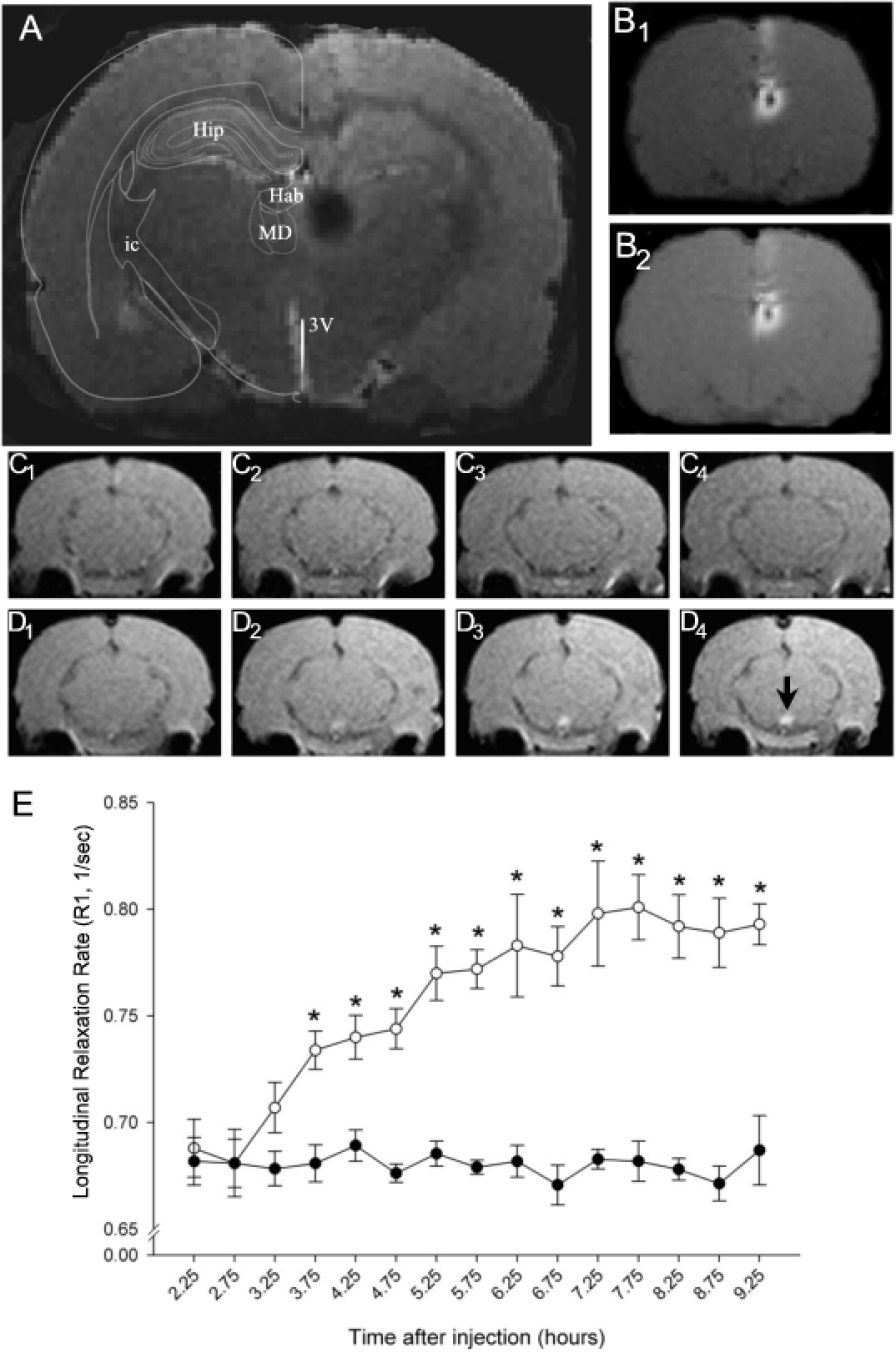
Time course of Mn^2+^ transport through the habenulomesencephalic pathway. (A) Anatomical MR image illustrating the site of unilateral infusion of MnCl_2_ into the dorsal diencephalon. The injection site, which appears as a black circle on the right side of the image, encompassed the entire habenula and a portion of the mediodorsal nucleus of the thalamus. An overlay adapted from the atlas of Paxinos and Watson [32] is positioned on the left side of the image for orientation. Abbreviations: Hip-hippocampus; Hab—habeunla; MD—mediodorsal nucleus of the thalamus; ic—internal capsule; 3V – 3^**rd**^ ventricle. (B) Corresponding T1 weighted images showing the distribution of Mn^**2+**^hyperintensityat the injection site 2.5 hours (B1) and 9 hours (B2) after infusion. (C-D) Representative coronal sections of the caudal midbrain at 0, 2, 4, and 6 hours (1–4, respectively) after the start of the imaging study in a sham control (C) and MnCl_2_-injected rat (D). Note the time-dependent increase in MR signal intensity in the IPN beginning 3.25 hours following the start of MnCl_2_ injection into the Hb (cf. region below black arrowhead in image D_4_). MRI images presented in A,B and D were obtained from the same rat. (E) Summary of the changes in longitudinal relaxation rate (ordinate) within the IPN as a function of the time (in hours) after MnCl_2_ injection in the Hb (abscissa). Each point represents the arithmetic mean ± SEM of 8–11 animals. Asterisks denote a significant difference from sham injection controls.

Microinjection of MnCl_2_ into the habenula resulted in a symmetrical region of signal enhancement encompassing both the medial and lateral component of the nucleus as well as the medial dorsal nucleus of the thalamus (Fig 1). Verification of the injection site was performed by comparing a high-resolution anatomical scan to the corresponding section from Paxinos and Watson [32] (Fig 1A). The injection site, which typically had a diameter in the coronal plane of < 1.5 mm at the start of the imaging session, did not change appreciably in size over the course of the next 6.5 hours (cf. Fig 1B_1_ and 1B_2_). A small but clearly visible area of low signal intensity was often observed at the center of the injection site and presumably represented an imaging artifact (e.g. intravoxel dephasing) associated with high MnCl_2_ concentration [33]. Consistent with this interpretation, we routinely observed a reduction in the size of the anomaly during the course of the experiment (cf. Fig 1B_1_ and 1B_2_).

**Fig 2:**
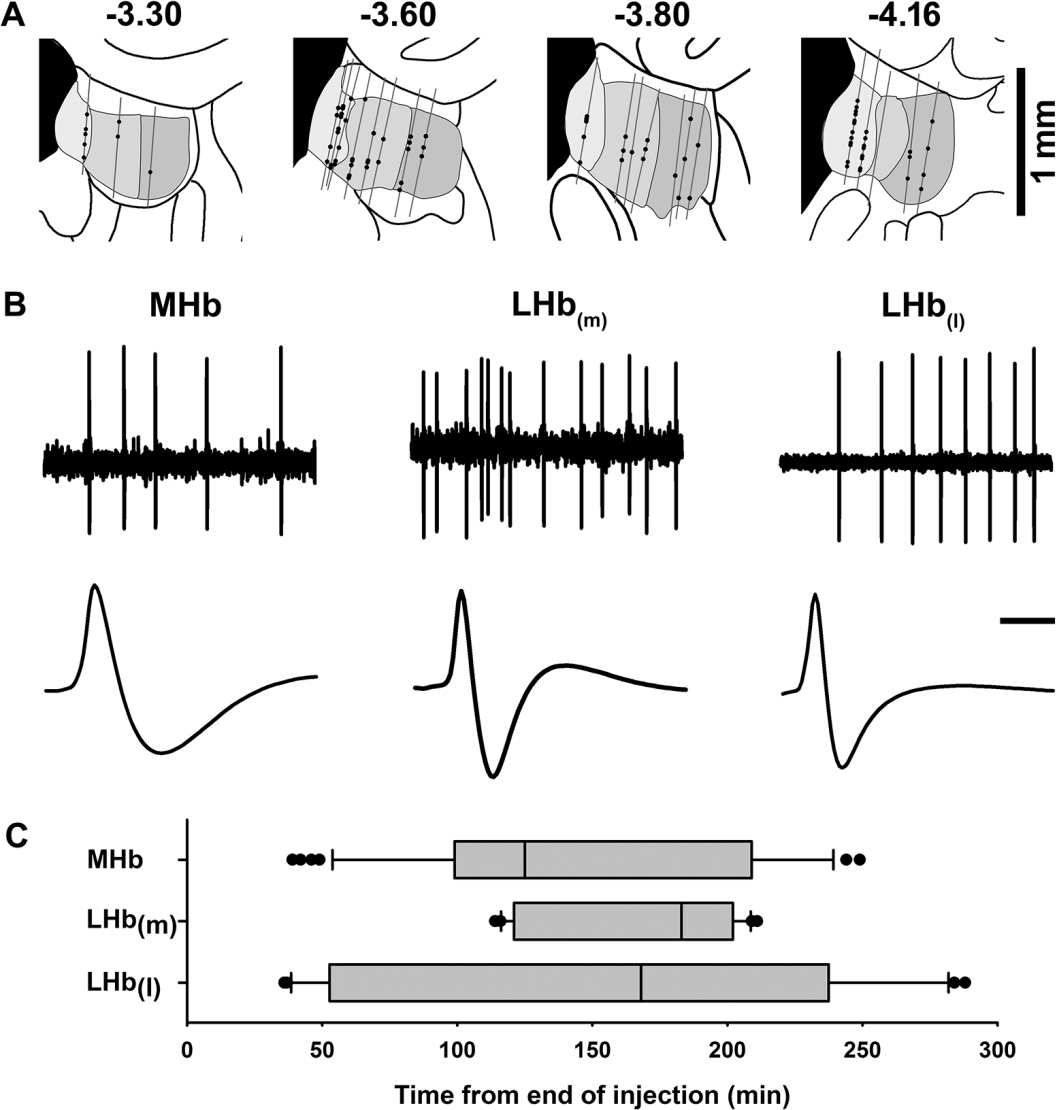
*In vivo* extracellular recordings of habenular neurons following intrahabenular MnCl_2_ injection. (A) Diagram of the anatomical location of cell tracks (gray lines) and recorded neurons (black circles) in coronal sections through the habenula. Grayed areas represent (from left to right) the MHb, LHb(m), and LHb(l). The anterioposterior position of each coronal section relative to bregma (in mm) is noted above each drawing. (B) Representative spike trains (upper traces, scale bar = 200 ms) and averaged waveforms (lower traces, scale bar = 1 ms) from spontaneously active neurons in each of the three subregions of the habenula. (C) Box and whisker plots showing the distribution of recording times from the end of MnCl_2_ injection for the MHb, LHb(m), and LHb(l). Each plot shows the median (vertical line within gray box), 25^**th**^ to 75^**th**^ percentile (gray box), 10^**th**^ to 90^**th**^ percentile (whiskers), and outliers (filled circles). Since track sequence was either medial to lateral or lateral to medial (see Methods for details), cells encountered within the LHb(m) tended to cluster near the middle of the recording period.

Previous studies have suggested that under some experimental circumstances, injection of MnCl_2_ is capable of temporarily suppressing neuronal excitability, possibly as a consequence of cationic hyperpolarization [34]. In an effort to determine whether MnCl_2_ had a similar effect on habenular neurons, extracellular single unit recording techniques were used to determine whether spontaneously firing neurons could be detected within the principal subdivisions of the habenula following its infusion. The number of spontaneously active cells in the MHb and the medial and lateral aspects of the lateral habenula, LHb(m) and LHb(l), respectively, were assessed in recordings obtained from 23 electrode tracks in 10 animals. Recordings began 20 minutes following the removal of the injection cannula and continued for up to 4.5 hours. The location of each track and the relative position of each spontaneously active neuron are illustrated in Fig 2A. Biphasic action potentials, organized into irregular single spike or bursting activity patterns (Fig 2B), were encountered throughout the recording session and showed no tendency to change in frequency over time (Fig 2C). The electrophysiological properties associated with habenular neurons varied by region (Table 1) including the number of cells encountered per track (ANOVA F_(2,20)_ = 9.3, p = 0.001), spontaneous firing rate (ANOVA F_(2,87)_ = 9.2, p = 0.0002) and spike duration (ANOVA F_(2,79)_ = 16.1, p < 0.0001). Post-hoc testing (Bonferroni corrected) revealed that of the three regions examined, the MHb had the highest incidence of spontaneously firing cells, differing significantly from both the lateral (p<0.01) and medial (p<0.01) divisions of the LHb. Spontaneously active MHb neurons also exhibited significantly longer duration action potentials than neurons in either the LHb(m) or LHb(l) (p< 0.01).

**Table 1:** Electrophysiological characteristics of spontaneously active habenula neurons following MnCl_2_ injection.

Region	Number of tracks	# Cells/track Mean ± SEM	Spontaneous Firing Rate, Hz (Pearson’s r, n)	Spike Width*, ms (Pearson’s r, n)
MHb	7	6.7 ± 1.1	5.5 ± 0.6	0.8 ± 0.05
			(0.14, 47)	(-0.03, 43)
LHb(m)	8	2.6 ± 0.7	15.9 ± 3.3	0.5 ± 0.05
			(0.30, 21)	(0.34, 19)
LHB (l)	8	2.8 ± 0.5	8.2 ± 2.1	0.4 ± 0.03
			(0.17, 22)	(0.06, 20)

All values represent the mean ± SEM. There were no significant correlations with time from injection (all p-values > 0.05).

* Spike width was measured at half-amplitude (peak-to-peak).

Neither firing rate nor spike width was significantly correlated with time from MnCl_2_ injection. The spontaneous firing rates of habenular neurons ranged from less than 1 to 50 Hz and tended to be higher in the LHb(m) than in either of the other two regions (P < 0.05).

Local Hb injection of MnCl_2_ resulted in a time-dependent increase in MRI contrast within the IPN as reflected by a significant increase in longitudinal relaxation rate (Fig 1C–1E). These changes are consistent with the hypothesized appearance of Mn^2+^ in terminals and axons of MHb projection neurons, which terminate exclusively within the IPN. The observed change in MRI contrast within the midbrain was restricted to the IPN (cf. Fig 1C_1_–1C_4_ and 1D_1_–1D_4_), although enhanced MRI signal was also detected in the region corresponding to the fasciculus retroflexus, the main pathway connecting the MHb with the IPN. Repeated measures analysis of variance (2x15 two-way ANOVA) of longitudinal relaxation rate obtained in a group of 8 MnCl_2_-injected rats and 7 vehicle control injected animals revealed main effects for treatment (F_(1,14)_ = 54.8, P < 0.001), time (F_(14,169)_ = 13.8, P < 0.001) and their interaction (F_(14,169)_ = 13.6, P < 0.001). Bonferroni corrected post-hoc testing indicated significant differences in R1 values between sham and MnCl_2_-injected rats beginning 3.75 hours following the start of the injection (Fig 1E).

**Fig 3:**
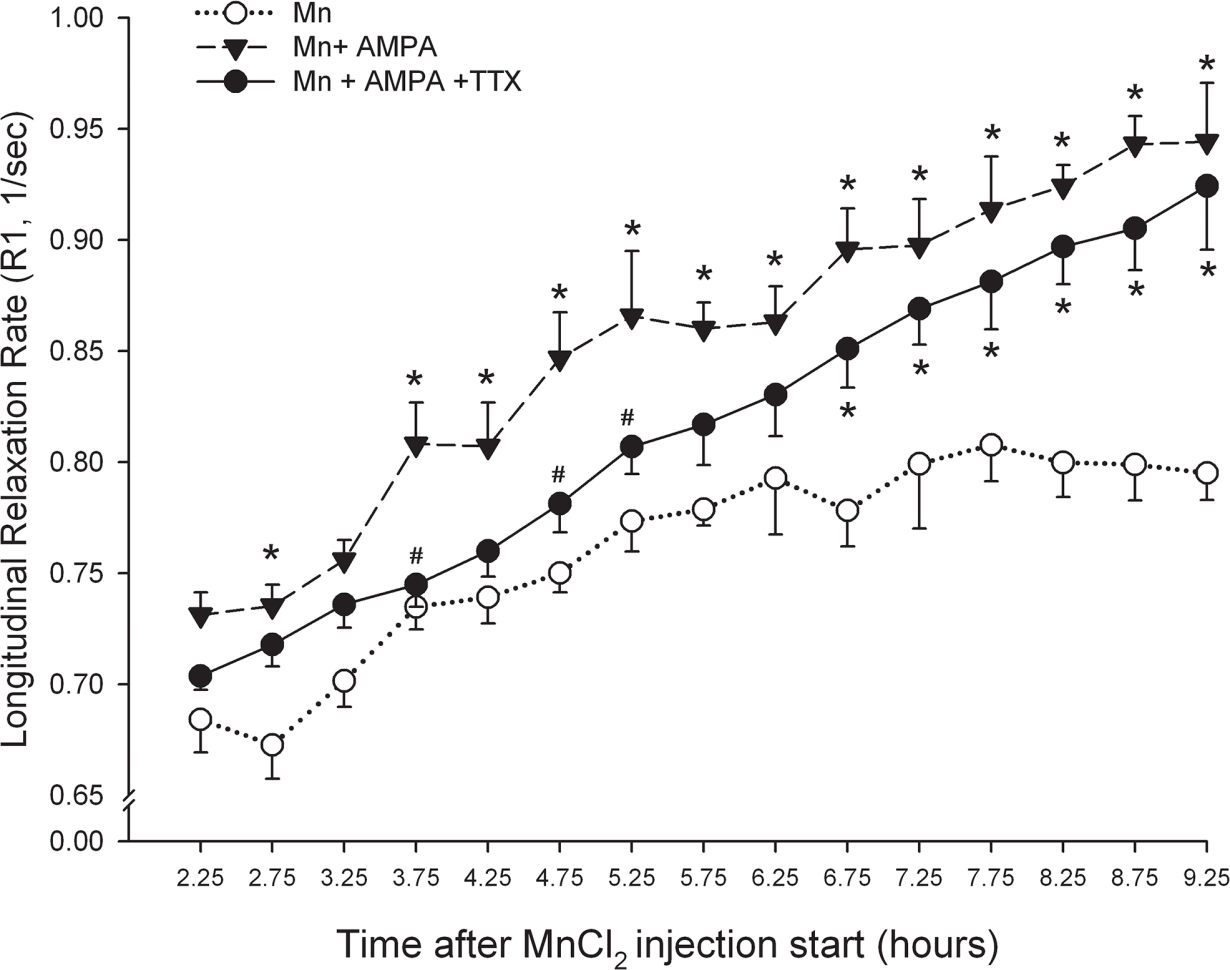
AMPA enhances Mn^2+^ uptake into Hb projection neurons in part through a TTX-insensitive mechanism. Changes in longitudinal relaxation rate in the IPN expressed as a function of time after the start of Mn^**2+**^ injection into the Hb. * P < 0.05 vs. Mn^**2+**^ only; # p< 0.05 vs. Mn^**2+**^ + AMPA.

In order to determine whether the accumulation of Mn^2+^ in the IPN was altered by changes in the excitability of MHb projection neurons, we compared R1 values in the IPN between groups of rats that received an injection of MnCl_2_ alone (n = 8) or in combination with the excitatory amino acid agonist, AMPA (n = 6). A third group of rats received MnCl_2_ together with AMPA and the fast sodium channel blocker, TTX (n = 8). Omnibus testing (3x15 two-way ANOVA) revealed significant main effects for treatment (F_(2,19)_ = 12.6, P < 0.001), time (F_(14,248)_ = 78.8, P < 0.001) and treatment x time interaction (F_(28,248)_ = 1.8, P < 0.05). As illustrated in Fig 3, addition of AMPA to the MnCl_2_ injection cocktail significantly increased the signal intensity within the IPN, consistent with an activity-dependent accumulation of Mn^2+^. Sustained differences in R1 values between MnCl_2_ and MnCl_2_ +AMPA treated rats began 3.75 hours after the start of the injection and continued for the duration of the experiment. Addition of TTX to the injection cocktail initially blocked AMPA-induced facilitation of Mn^2+^ signaling in the IPN (Fig 3). However, these effects dissipated over time and by ~ 6.5 hours post-injection, relaxation rates within the Mn+AMPA+ TTX group, while still lower than values obtained in rats that received Mn+AMPA, differed significantly from Mn^2+^ only control group.

**Fig 4:**
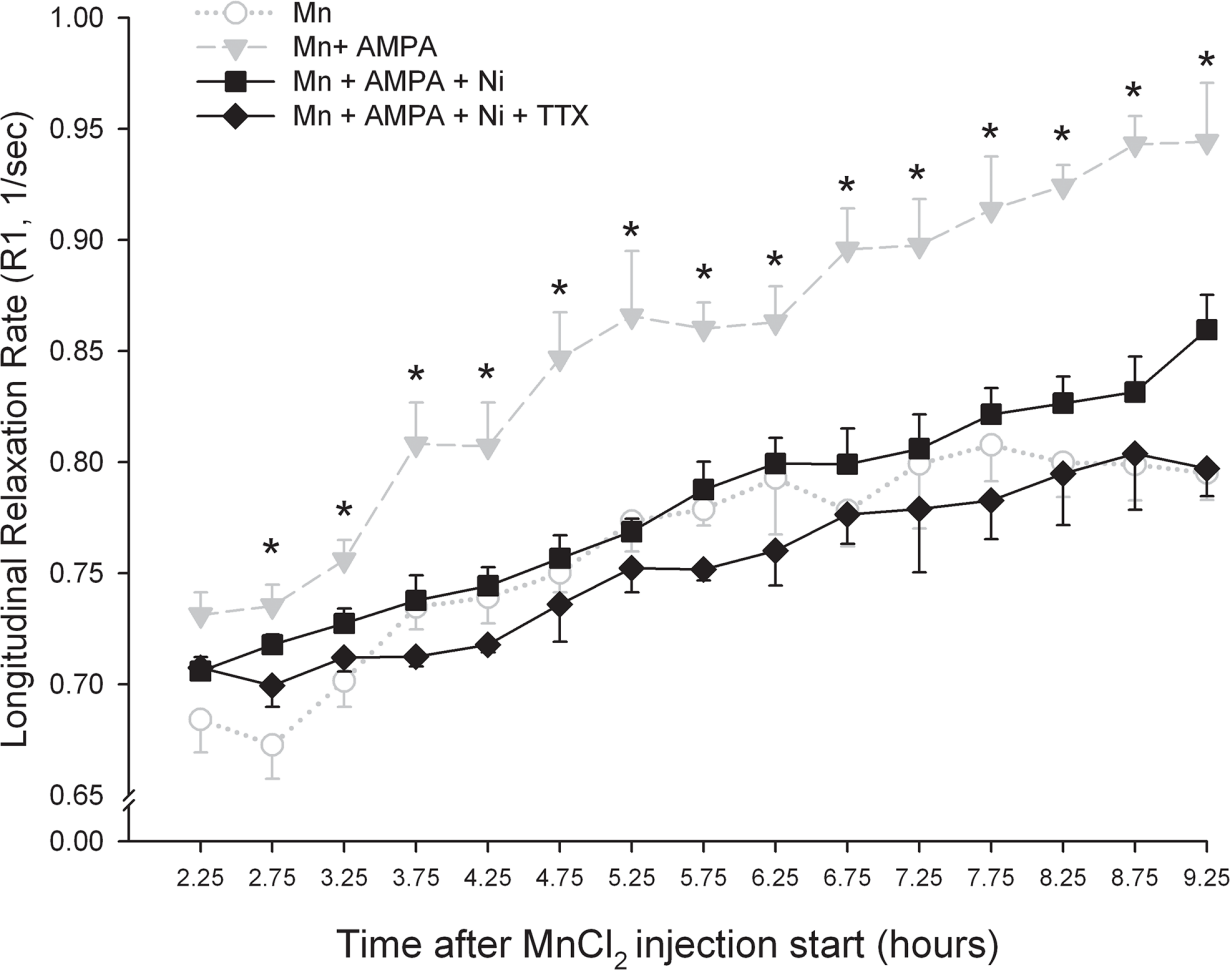
Concomitant blockade of voltage-activated Na^+^ and Ca^2+^ channels prevents AMPA-induced enhancement of Mn^2+^ accumulation in the IPN. Changes in longitudinal relaxation rate in the IPN expressed as a function of time after the start of Mn^**2+**^ injection into the Hb. * P < 0.05 vs. Mn^**2+**^ only.

In an effort to determine whether Mn^2+^ uptake was Ca^2+^ dependent, we assessed the effects of the broad-spectrum Ca^2+^ channel blocker, NiCl_2_ on the AMPA-induced potentiation of Mn^2+^ signaling in the IPN. In order to ensure a complete block of voltage-activated Ca^2+^ channels, NiCl_2_ was injected into the habenula 40 minutes prior to subsequent injection of the AMPA+MnCl_2_ cocktail. Omnibus testing (4x15 two-way ANOVA) revealed significant main effects for treatment (F_(3,18)_ = 14.8, P < 0.001), time (F_(14,230)_ = 58.9, P < 0.001), and a treatment x time interaction (F_(42,230)_ = 2.3, P < 0.001). As illustrated in Fig 4, pretreatment with NiCl_2_ prevented the time-dependent enhancement in R1 induced by AMPA. Addition of TTX to the injection cocktail resulted in a trend toward a further reduction in signal enhancement, however neither group differed significantly from Mn^2+^ alone.

## Discussion

An increasing number of imaging studies have sought to exploit the ability of Mn^2+^ to penetrate voltage-activated Ca^2+^ channels as a means of visualizing activity dependent changes in specific brain circuits. In the present study, we tested this presumption by co-administering Mn^2+^ with drugs known to modulate neuronal excitability. Our results support the proposition that enhanced neuronal activity facilitates Mn^2+^ uptake in a sodium and Ca^2+^-dependent fashion. Surprisingly however, basal rates of Mn^2+^ uptake by habenular projection neurons were not altered by changes in neuronal excitability and may have been driven by a second independent Mn^2+^ transport mechanism.

### The Habenulomesencephalic Pathway—Methodological Considerations

The highly circumscribed habenulomesencephalic pathway is particularly well suited for assessing the role of neuronal activity on axonal transport of Mn^2+^ using MRI. The habenula consists of a bilaterally symmetrical pair of nuclei comprised of medial and lateral subdivisions. Efferents from the medial habenula travel within the core of the fasciculus retroflexus and terminate exclusively within the IPN [35], a midline structure readily identifiable in MR images. By contrast, axons arising from neurons in the lateral habenula travel in the mantle of the fasciculus retroflexus and innervate regions medial and caudal to the IPN including the ventral tegmental area, rostromedial tegmental area and the dorsal and median raphe [23]. The scarcity of ascending habenular projections combined with the aggregation of efferent axons from a bilateral structure within a fiber bundle that converges on a single centrally-located midline region provides an ideal anatomical substrate to quantify changes in Mn^2+^ uptake and transport by CNS neurons using MEMRI.

In the present study, MnCl_2_ was delivered directly into the habenular parenchyma by stereotaxic microinjection. Although used extensively in rodent MEMRI studies, local injection of MnCl_2_ into the brain can result in neurotoxicity attributable to the direct effect of Mn^2+^ on cellular processes as well as non-specific vehicle-related effects [12]. Systematic study of the acute neurotoxic effects associated with parenchymal MnCl_2_ injection revealed that the threshold for eliciting gliosis and neuronal cell death in the rat cortex is 8 and 16 nmol, respectively [24]. In the present study, the concentration of Mn^2+^ in the habenula was 3.15 nmol, well below that needed to induce neurotoxic changes in brain tissue. The vehicle buffer system, pH, and osmolality were optimized to eliminate the physicochemical toxicity of the MnCl_2_ solution.

Despite the precautions taken to avoid neuronal toxicity, the possibility that Mn^2+^ altered neuronal activity within the habenula though another mechanism could not be excluded. For example, visual evoked potentials in the optic nerve are suppressed for up to 4 hours following relatively low concentrations of Mn^2+^ (6.25 nmol) potentially through a process involving cation-induced membrane hyperpolarization [34]. However, a parallel series of extracellular recordings obtained from the habenula following MnCl_2_ injection revealed the presence of spontaneously firing neurons in each of its principal subdivisions. The incidence of spontaneously firing cells did not change during the course of the recording session and there was no evidence of a time-dependent change in action potential duration or spontaneous firing rate. Action potentials were free from injury artifacts and the range of firing rates similar to recordings obtained from habenular neurons under anesthetic conditions similar to those used in the present study [36, 37]. The interval over which these recordings were conducted bracketed the time required for the MEMRI signal to reach the IPN (~ 3.5 hrs) corresponding to a transport rate of approximately 1.7 mm/hour and commensurate with the rate of fast axonal transport [38]. Collectively, these data indicate that MnCl_2_ injection did not exert an acute or delayed toxic effect on the physiological properties of habenular neurons supporting the proposition that the increase in MRI signal intensity in the region of the IPN occurs as consequence of Mn^2+^ transport by Hb projection neurons.

Injection sites typically included the entire left habenula as well as a portion of the medial dorsal thalamus and showed little variation in size between subjects. Spread into the underlying thalamic region was not considered confounding as this area is not known to project directly to the IPN [39]. Despite encompassing both the medial and lateral aspects of the habenula, the enhanced MRI signal was restricted to the IPN, a structure that is innervated exclusively by neurons within the MHb. Several factors likely contributed to this. First, unlike MHb efferents, LHb neurons project to several brain regions including the PAG, dorsal and ventral raphe, VTA, and RMTg likely resulting in a substantial reduction of the MEMRI signal intensity at any one region. Second, neurons comprising the habenulointerpeduncular tract do not exhibit the typical reduction in metabolic activity seen during anesthesia [40, 41] but instead exhibit *increased* glucose utilization [42–45]. This could have resulted in greater uptake and faster axonal transport of Mn^2+^ within the habenulointerpeduncular tract than in projections arising from the lateral habenula which show the typical depression in glucose metabolism during anesthesia[43]. Finally, our results indicate that the proportion of spontaneously active neurons in the MHb following MnCl_2_ infusion is significantly greater than in either subdivision of the LHb, supporting the implicit proposition that the strength of the Mn^2+^ signal corresponds to the rate of neuronal firing. Collectively, these data may explain the robust labeling of the habenulopeduncular tract following systemic administration or intracerebro-ventricular microinjection of MnCl_2_ [46].

### Activity-Dependent Mn^2+^ transport—Role of Spike-Dependent Mechanisms

Excitatory amino acid receptors are likely to play a key role in activity-dependent MEMRI both as a source of depolarizing current underlying the activation of voltage-gated Ca^2+^ channels and as a potential Mn^2+^ ionophore. Neurons in both the medial and lateral habenula have been shown to express functional AMPA-type excitatory amino acid receptors [47–50]. In the present study, co-infusion of AMPA together with Mn^2+^ significantly increased R1 values in the IPN compared to Mn^2+^ alone, changes that presumably reflect activity-dependent increases in Mn^2+^ entry into MHb projection neurons. AMPA receptors containing GluR-A,-C and-D subunits exhibit high Ca^2+^ permeability and could theoretically provide a conduit for Mn^2+^ entry into CNS neurons [51–53]. However, AMPA currents in MHb neurons show a distinctly linear current-voltage relationship indicative of a receptor with low Ca^2+^ permeability [50]. In addition, these receptors are characterized by a rapid deactivation and desensitization and thus do not show sustained currents even during prolonged exposure to agonist. Together, these data suggest that Mn^2+^ entry through canonical voltage-activated Ca2+ channels activated by AMPA-induced membrane depolarization underlies the enhanced MEMRI signal intensity within the IPN.

In an effort to determine whether AMPA-induced increases in neuronal spiking contributed to enhanced Mn^2+^ uptake by MHb projection neurons, we first assessed the effects of TTX on the AMPA-induced increase in R1 values within the IPN. Addition of the fast sodium channel pore blocker initially occluded AMPA-induced increases in IPN signal intensity consistent with its well known ability to block spontaneous action potentials when injected locally into the brain [54–56]. The effects of TTX began to dissipate approximately 6 hours after injection, likely as a result of recovery from blockade of fast sodium channels and a resumption of spontaneous spiking. Further support for activity-dependent Mn2+ uptake in general and the specific contribution of voltage-activated Ca^2+^ channels in this process was provided by results showing that Ni^2+^ significantly reduced the AMPA-driven enhancement of Mn^2+^ signal intensity in the IPN. Unlike Mn^2+^ which permeates Ca^2+^ channels, Ni^2+^ is a potent Ca^2+^ channel pore blocker with preferential affinity for T-type Ca^2+^ channels [57, 58]. Notably, T-type Ca^2+^ channels are responsible for the low threshold Ca^2+^ spikes exhibited by MHb neurons [59]. Addition of TTX to the Ni^2+^-containing cocktail resulted in a trend toward further reduction in IPN signal intensity, further supporting the role for impulse-dependent uptake of Mn^2+^ uptake into MHb neurons.

### Activity-Independent Mn^2+^ Uptake

While blockade of fast sodium and voltage-dependentcalcium channels occluded AMPA-induced increases in Mn^2+^ uptake, a consistent finding throughout this study was the failure of either drug, alone or in combination, to prevent Mn^2+^ uptake by habenular neurons under basal (unstimulated) conditions. The inability of TTX and Ni^2+^ to even *attenuate* MEMRI intensity in the IPN under basal conditions would seem to suggest that its entry into MHb projection neurons does not occur exclusively through activity-dependent mechanisms. Activity-*independent* transport of Mn^2+^ has been convincingly documented within the mouse retinotectal pathway [22, 34] where local infusion of TTX was ineffective in attenuating Mn^2+^ uptake by retinal ganglion cells. Mn^2+^ entry through heavy metal transporters [2], provide a potential explanation for these results. The range of Mn^2+^ concentrations typically used for MEMRI studies can disrupt heavy metal homeostasis in mice [60]. With respect to the present findings, it is interesting to note that neurons in the MHb have the highest density of transferrin receptor protein in the brain [61, 62].

## Conclusions

The results of the present study suggest that uptake of Mn^2+^ by MHb neurons comprising the habenulointerpeduncular pathway, occurs through activity-dependent and activity-independent mechanisms. In the anesthetized rat under basal conditions, uptake appears to occur at least in part, through an impulse-independent process possibly involving heavy metal transporters. However, when MHb neurons are excited by activation of AMPA receptors, Mn^2+^ uptake is enhanced through an activity-dependent mechanism that is sensitive to Na^+^ and Ca^2+^ channel blockade. These results suggest that Mn^2+^ uptake and transport by at least some CNS neurons may not invariably reflect the state of neuronal excitability and point to the importance of considering passive Mn uptake mechanisms when interpreting MEMRI results.
